# Subclinical Myocardial Dysfunction in Patients with Persistent Dyspnea One Year after COVID-19—Why Should Screening for Cardiovascular Diseases Be Performed? Reply to Vankrunkelsven, P. Tendentious Paper—Titles and Wrong Conclusions Lead to Fear in the Population and Medical Overconsumption. Comment on “Luchian et al. Subclinical Myocardial Dysfunction in Patients with Persistent Dyspnea One Year after COVID-19. *Diagnostics* 2022, *12*, 57”

**DOI:** 10.3390/diagnostics12081837

**Published:** 2022-07-29

**Authors:** Maria-Luiza Luchian, Andreea Motoc, Stijn Lochy, Julien Magne, Dries Belsack, Johan De Mey, Bram Roosens, Karen Van den Bussche, Sven Boeckstaens, Hadischat Chameleva, Jolien Geers, Laura Houard, Tom De Potter, Sabine Allard, Caroline Weytjens, Steven Droogmans, Bernard Cosyns

**Affiliations:** 1 Department of Cardiology, Centrum voor Hart-en Vaatziekten, Universitair Ziekenhuis Brussel, Vrije Universiteit Brussel (VUB), Laarbeeklaan 101, 1090 Brussels, Belgium; andreeaiulia.motoc@uzbrussel.be (A.M.); stijn.lochy@uzbrussel.be (S.L.); bram.roosens@uzbrussel.be (B.R.); karen.vandenbussche@uzbrussel.be (K.V.d.B.); sven.boeckstaens@uzbrussel.be (S.B.); jolien.geers@uzbrussel.be (J.G.); laura.houard@uzbrussel.be (L.H.); caroline.weytjens@uzbrussel.be (C.W.); steven.droogmans@uzbrussel.be (S.D.); bernard.cosyns@uzbrussel.be (B.C.); 2 CHU Limoges, Hôpital Dupuytren, Service Cardiologie, 87042 Limoges, France; julien.magne@unilim.fr; 3 Faculté de Médecine de Limoges, 2, rue Marcland, 87000 Limoges, France; 4 Department of Radiology, Universitair Ziekenhuis Brussel, Vrije Universiteit Brussel (VUB), Laarbeeklaan 101, 1090 Brussels, Belgium; dries.belsack@uzbrussel.be (D.B.); johan.demey@uzbrussel.be (J.D.M.); 5 Faculty of Medicine and Pharmacy, Vrije Universiteit Brussel (VUB), Laarbeeklaan 103, 1090 Brussels, Belgium; hadischat.chameleva@uzbrussel.be (H.C.); tom.de.potter@vub.be (T.D.P.); 6 Department of Internal Medicine and Infectious Diseases, Universitair Ziekenhuis Brussel, Vrije Universiteit Brussel (VUB), Laarbeeklaan 101, 1090 Brussels, Belgium; sabine.allard@uzbrussel.be

We have read with interest the comment by Vankrunkelsven P. [1] on the results of our study emphasizing the subtle cardiac changes attributed to SARS-CoV-2 infection following an acute episode of COVID-19 pneumonia. A total of 66 COVID-19 patients without a previous history of cardiorespiratory diseases were evaluated at 12 months for potential subclinical cardiac dysfunction following the acute infection [2].

The presence of persistent dyspnea was observed in one-third of the recovered COVID-19 patients, in line with the current data on the long-term evolution of COVID-19 [3,4]. Our study sought to gain a more detailed insight into the cardiac mechanics potentially associated with these symptoms. We report a significant association between the presence of dyspnea at one year following COVID-19 and a reduction in global constructive work (GCW) and global work index (GWI) parameters, suggesting subtle cardiac abnormalities as a potential substrate for the ongoing symptoms in discharged COVID-19 patients [2]. Although the absolute values of the two parameters were in the so-called “normal range”, there were significant differences between COVID-19 patients with and without dyspnea at one-year follow-up. Hereby, it is important to emphasize that all the patients have had COVID-19, in contrast to the reference values derived from the healthy study subjects of the NORRE study [5]. Our results are further supported by recent data showing the presence of endothelial dysfunction and persistent oxidative burden in symptomatic discharged COVID-19 patients linked to a mildly compromised cardiac performance, indicated by the values of myocardial work parameters and left ventricle global longitudinal strain [6,7]. The echocardiographic parameters at t 12-month follow-up reported by Ikonomidis et al, similar to our results, remained within the “normal range”; however, they were significantly different when compared to healthy individuals [6].

In the study by D’ Andrea A et al, cited by Vankrunkelsven P., global longitudinal strain (GLS) was reduced in patients with proven COVID-19 myocarditis [8]. Hereby, GLS improved after 6 months, but it remained abnormal compared to the control group, outlining the importance of a cardiac follow-up. 

Moreover, a recent report by Xie Y. et al showed a substantial increase in cardiovascular disease burden, e.g., ischemic heart diseases, heart failure, and arrhythmia, both in hospitalized and non-hospitalized COVID-19 patients, irrespective of age, race, and other cardiovascular risk factors, outlining the necessity of specific medical management strategies targeting cardiovascular health and diseases [9]. Long COVID, a newly emerged condition characterized by the persistence of several symptoms, including dyspnea, has important reverberations on patients’ quality of life. The pathophysiological mechanisms behind the evolution to chronic COVID-19 are still under investigation, with more data showing a combination of metabolic dysregulation, chronic inflammation, and endothelial dysfunction with significant multiorgan impact, including on the cardiovascular system [10], as previously suggested by the results of the present study.

In conclusion, COVID-19 remains a complex disease that may have long-term consequences, including cardiovascular ones. At first sight, the global standard echocardiographic evaluation appears preserved after one year, reassuring the absence of major abnormalities of the cardiac function. However, subtle cardiac changes may be present and linked to the ongoing symptoms. Considering the rise in the number of patients recovering from COVID-19 and developing ‘Long COVID’, a stepwise approach with an integrated cardiac follow-up remains crucial for the early detection of potential cardiac abnormalities. This will help to gain further objective insight into this new disease we are confronted with. This has nothing to do with increasing fear in the population or medical overconsumption, as stated by Vankrunkelsven P.
